# Ectopic MicroRNA-150-5p Transcription Sensitizes Glucocorticoid Therapy Response in MM1S Multiple Myeloma Cells but Fails to Overcome Hormone Therapy Resistance in MM1R Cells

**DOI:** 10.1371/journal.pone.0113842

**Published:** 2014-12-04

**Authors:** Ajay Palagani, Ken Op de Beeck, Stefan Naulaerts, Jolien Diddens, Chandra Sekhar Chirumamilla, Guy Van Camp, Kris Laukens, Karen Heyninck, Sarah Gerlo, Pieter Mestdagh, Joke Vandesompele, Wim Vanden Berghe

**Affiliations:** 1 Laboratory of Protein Chemistry, Proteomics and Epigenetic Signalling (PPES), Department of Biomedical Sciences, University of Antwerp (UA), Antwerp, Belgium; 2 Laboratory of Eukaryotic Gene Expression and Signal Transduction (LEGEST), Department of Physiology, Ghent University, Ghent, Belgium; 3 Center of Medical Genetics, Department of Biomedical Sciences, University of Antwerp, Antwerp, Belgium; 4 Laboratory of Cancer Research and Clinical Oncology, Department of Medical Oncology, University of Antwerp/Antwerp University Hospital, Antwerp, Belgium; 5 Biomedical Informatics Research Center Antwerp (Biomina), University of Antwerp & University Hospital Antwerp, Antwerp, Belgium; 6 Advanced Database Research and Modelling (ADReM), Department of Mathematics & Computer sciences, University of Antwerp (UA), Antwerp, Belgium; 7 VIB-UGent Department of Medical Protein Research, Ghent, Belgium; 8 Center for Medical Genetics, Ghent University Hospital, Ghent, Belgium; University of Ulm, Germany

## Abstract

Glucocorticoids (GCs) selectively trigger cell death in the multiple myeloma cell line MM1S which express NR3C1/Glucocorticoid Receptor (GR) protein, but fail to kill MM1R cells which lack GR protein. Given recent demonstrations of altered microRNA profiles in a diverse range of haematological malignancies and drug resistance, we characterized GC inducible mRNA and microRNA transcription profiles in GC sensitive MM1S as compared to GC resistant MM1R cells. Transcriptome analysis revealed that GCs regulate expression of multiple genes involved in cell cycle control, cell organization, cell death and immunological disease in MM1S cells, which remain unaffected in MM1R cells. With respect to microRNAs, mir-150-5p was identified as the most time persistent GC regulated microRNA, out of 5 QPCR validated microRNAs (mir-26b, mir-125a-5p, mir-146-5p, mir-150-5p, and mir-184), which are GC inducible in MM1S but not in MM1R cells. Functional studies further revealed that ectopic transfection of a synthetic mir-150-5p mimics GR dependent gene expression changes involved in cell death and cell proliferation pathways. Remarkably, despite the gene expression changes observed, overexpression of mir-150-5p in absence of GCs did not trigger significant cytotoxicity in MM1S or MM1R cells. This suggests the requirement of additional steps in GC induced cell death, which can not be mimicked by mir-150-5p overexpression alone. Interestingly, a combination of mir-150-5p transfection with low doses GC in MM1S cells was found to sensitize therapy response, whereas opposite effects could be observed with a mir-150-5p specific antagomir. Although mir-150-5p overexpression did not substantially change GR expression levels, it was found that mir-150-5p evokes GR specific effects through indirect mRNA regulation of GR interacting transcription factors and hormone receptors, GR chaperones, as well as various effectors of unfolded protein stress and chemokine signalling. Altogether GC-inducible mir-150-5p adds another level of regulation to GC specific therapeutic responses in multiple myeloma.

## Introduction

Multiple myeloma (MM) is a B-cell neoplasm characterized by the accumulation of clonal malignant plasma cells in the bone marrow and often correlated with various cytogenetic abnormalities such as del(13), t(11∶14), non-hyperdiploidy, and del(17p) [1], [2]. The disease accounts for 10% of the haematological malignancies and approximately 1% of cancer-related deaths in Western countries [3]. Therapy against multiple myeloma consists of drugs which can decrease the clonal plasma cell population. Initial treatment towards the disease depends mainly on patients age and comorbidities. The ability of glucocorticoids (GCs) to efficiently kill lymphoid cells has led to their inclusion in essentially all chemotherapy protocols for lymphoid malignancies. For patients under the age of 65 high doses of chemotherapy of different combinations such as thalidomide–dexamethasone-bortezomib based regimens, and lenalidomide–dexamethasone followed by autologous haematopoietic stem cell transplantation has been a practice in the clinic in the recent years [4], [5], [6], [7], [8]. Despite the progress in therapy, MM remains largely incurable, due to low remission rates of conventional therapies resulting in short survival times (3–4 years) and the development of drug resistance. Numerous novel drug combinations are currently being tested to prevent resistance and improve GC efficacy in the therapy of lymphoid malignancies [9].

Glucocorticoids (GCs) are steroid hormones, which exert their pro- or anti-apoptotic actions via the glucocorticoid receptor (GR, NR3C1) and alterations in gene expression [10], [11]. The GRα isoform, which is the main mediator of GC effects, comprises three main functional domains: an N-terminal domain (NTD), a DNA binding domain (DBD) and a ligand binding domain (LBD) [12]. The inactive form of GRα primarily resides in the cytoplasm forming a chaperone complex with its binding partners such as heat shock proteins, FKBP51, FKBP52, CYP40 and other proteins [13]. Upon binding with the GC’s, the GR becomes activated and translocates to the nucleus. The activated GR has several different modes of action at the cellular level. The most prominent one is transactivation in which the activated GR binds to a glucocorticoid-response element (GRE) and induces gene expression. Further, GR can bind negative GRE sites (nGREs) or tether bound transcription factors (NFκB, AP1, P53 or STAT) and hence (trans)represses gene expression [14]. Although being known as a strong inducer of apoptosis in lymphoid cells for almost a century, the signaling pathways regulating the susceptibility or resistance of cancer cells to GCs are only partly revealed. Besides slow genomic GC actions depending on nuclear gene regulation, also rapid non-genomic effects of GCs in the cytoplasm modulate the apoptotic response [15], [16]. With respect to development of GC therapy resistance, various mechanisms have been proposed including GR degradation, Akt dependent GR phosphorylation, reduced histone deacetylase-2 (HDAC2) activity, excessive activation of the transcription factor NFkB or AP1, elevated macrophage migration inhibitory factor, oxidative stress and P-glycoprotein mediated regulation of drug efflux [17], [18], [19], [20]. A novel emerging hypothesis is that microRNAs modulate GC therapy response via (post-transcriptional) regulation of multiple mRNA targets involved in GR signaling related to cell proliferation and cell death [21], [22], [23], [24]. MiRNAs are short non-coding RNAs of approximately 22 nucleotides length which regulate expression of tens to hundreds of genes via mRNA degradation or translational repression, based on the sequence complementarity to the miRNA seed region (positions 2–8) at the 3′UTR of the mRNA [25]. Alternatively, miRNAs may indirectly reprogram transcriptional responses by changing mRNA levels of transcription factor levels [26]. As such, microRNAs may represent a possible feedback mechanism to fine-tune the magnitude of GC induced cell death via direct regulation of GR mRNA/protein levels or indirect regulation of GR interaction partners or downstream GC/GR responsive target mRNAs. Since miRNA dysregulation has a profound impact on cell differentiation, development, apoptosis and cell cycle progression, their expression profiles can be used as biomarkers for cancer type and grade (prognosis) and/or personalized therapy response [27]. To characterize the regulatory role of GC inducible microRNAs in multiple myeloma therapy response, we have compared mRNA and microRNA transcription profiles in GC sensitive MM1S cells and GC resistant MM1R cells upon exposure to GC. The latter cell model is frequently used for elucidation of regulatory mechanisms involved in GC therapy resistance in MM cells [28].

## Materials and Methods

### Cell Culture and cell viability

GC sensitive MM1S (CRL-2974) and GC resistant MM1R cell lines (CRL-2975) have been previously described [28] and were purchased from ATCC. Briefly, MM1S cells are a B-lymphoblast myeloma cell line derived from a 42 years old black female. The MM1R cell line is a steroid resistant subclone derived from MM1S cells, chronically exposed to dexamethasone (Dex). Cells were cultivated in RPMI-1640 Medium supplied with 10% Fetal Bovine Serum (E.U Approved; South American Origin) and 0.5% Penicillin/Streptomycin Solution (Invitrogen). The cell lines were additionally supplemented with 1% MEM Non-Essential Amino Acids and 1% Sodium Pyruvate (Invitrogen). Each cell line was maintained at 37°C in the atmosphere of 5% CO_2_ and 95% air, 95–98% humidity, until confluence. Cell viability was assessed by using colorimetric assay with 3-(4, 5-dimethylthiozol-2-yl)-2, 5-diphenyltetrazolium bromide (MTT) (Sigma Aldrich, St. Louis, MO, United States of America) as previously described [29].

### Antibodies and reagents

Water soluble dexamethasone was purchased from (Sigma Aldrich, St. Louis, MO, United States of America) and 10 mM stock solutions were prepared in sterile water. Anti-GRα (sc-8992) antibody, anti-PARP antibody (sc-7150) were purchased from Santa Cruz Biotechnology (Dallas, United States of America), and anti-α-tubulin (T5168) was purchased from Sigma Aldrich (St. Louis, MO, US).

### RNA extraction and microarray processing

Total RNA from MM1S and MM1R cells, either Dex treated (1 µM) or transfected with mock or mir-150-5p mimetics, from three independent experiments was isolated using RNeasy Mini Kit (Qiagen, Venlo, Netherlands) according to the manufacturer’s protocol. Following extraction and concentration measurement (NanoDrop 1000, Thermo Scientific, Waltham, MA, USA) total RNA was quality controlled on a Bio-Rad Experion (Bio-Rad, Hercules, CA, USA). 500 ng of total RNA was amplified using the Illumina Total Prep RNA Amplification kit (Life Technologies, Carlsbad, CA, USA). Briefly, RNA was reverse transcribed using T7 oligo(dT) primers, after which biotinylated cRNA was synthesized through an in vitro transcription reaction. 750 ng of amplified cRNA was hybridized to a corresponding array of a HumanHT12 beadchip (Illumina, San Diego, CA, USA). Multiplicates were performed. The beadchip was incubated for 18 hours at 58°C in a hybridization oven under continuous rocking. After several consecutive washing steps (see manufacturer’s protocol), bead intensities were read on an Illumina Iscan.

### Microarray data analysis

All the raw data intensities generated were read using the “beadarray” package (v2.8.1) in R [30]. Intensities were quantile normalized and differential gene expression between samples was estimated using “Limma” (v3.14.1). Resulting p-values were corrected for multiple hypothesis testing using the Benjamini-Hochberg method. Next to estimating gene expressions, euclidean distances between samples and genes were calculated and used as a distance metric in a hierarchical cluster analysis with MeV [31]. Pathway analysis was performed in the Ingenuity Pathway Knowledge Base (Ingenuity Systems, Redwood City, CA, USA) according to the instructions provided. A fold change cut-off of 2 as well as false discovery rate of 0.1% were set to identify genes whose expression was significantly differentially regulated. Raw array data were uploaded to the Gene Expression Omnibus (GEO) database and have accession number GSE59805.

### cDNA conversion and QPCR analysis

Briefly 1 µg of RNA was converted into cDNA using Go Script reverse transcription system according to manufacturer’s instructions (Promega, Madison, WI, United States of America). Sybr green was purchased from (Bioline, London, UK). QPCR was performed using ABI7300 system (Applied biosystems, Life Technologies Corporation).

### miRNA analysis

miRNA isolation was performed with a miRNeasy kit (Qiagen, Venlo, Netherlands) according to manufacturer instructions. Stem-loop RT-qPCR miRNA expression profiling of 760 microRNAs was done as previously described [32], [33]. Raw expression values were normalized using the global mean normalization strategy [32]. Validation of QPCR array results was done by reverse transcription of total RNA containing miRNA and miRNA detection by real-time PCR, using respectively miScript II RT Kit and miScript SYBR Green PCR Kit (Qiagen, Venlo, Netherlands). MicroRNA specific QPCR primer sets against human mir-146a (MS00003535), mir-491-5p (MS00031899), mir-146b-5p (MS00003542), mir-26b-5p (MS00003234), mir-150-5p (MS00003577), mir-330-3p (MS00031738), mir-195 (MS00003703), mir-184(MS00003640), mir-125a-5p (MS00003423) were purchased from Qiagen (Venlo, Netherlands). For microRNA target and pathway prediction, miRWalk software was applied [34]. Correlation of mRNA and microRNA transcription profiles of MM1S and MM1R cells either non-treated or treated with Dex, or following mock transfection or transfection with mir-150-5p mimetic was performed with IPA software.

### In vitro transfection of MM cells with synthetic miR-150-5p mimetic and antagomir

Mir-150-5p mimetic and antagomir were purchased from Qiagen (Venlo, Netherlands). A total of 5×10^6^ cells was electroporated with mock control or synthetic mir-150-5p at a final concentration of 150 nmol/L, with Nucleofector Kit V, using AMAXA Transfection System, program O23 from (LONZA, Basel, Switzerland) as described elsewhere [35].

### Protein isolation

5×10^6^ cells were seeded in 10 cm dishes and protein lysates of different experimental setups were prepared for Western analysis with 1XSDS Sample buffer containing 62.5 mM Tris-HCL pH-6.8, 2% SDS, 10% Glycerol, 0.5% Bromophenol Blue, DTT (5 mM).

### Statistical Analysis

Statistics were performed using one way ANOVA, two way ANOVA followed by Dunnett’s and Bonferroni post-test and one-tailed paired student t-test using graph pad prism5 software.

## Results

### Dexamethasone decreases cell viability in a dose and time dependent manner in GC sensitive MM1S but not GC resistant MM1R cells

**Figure 1:**
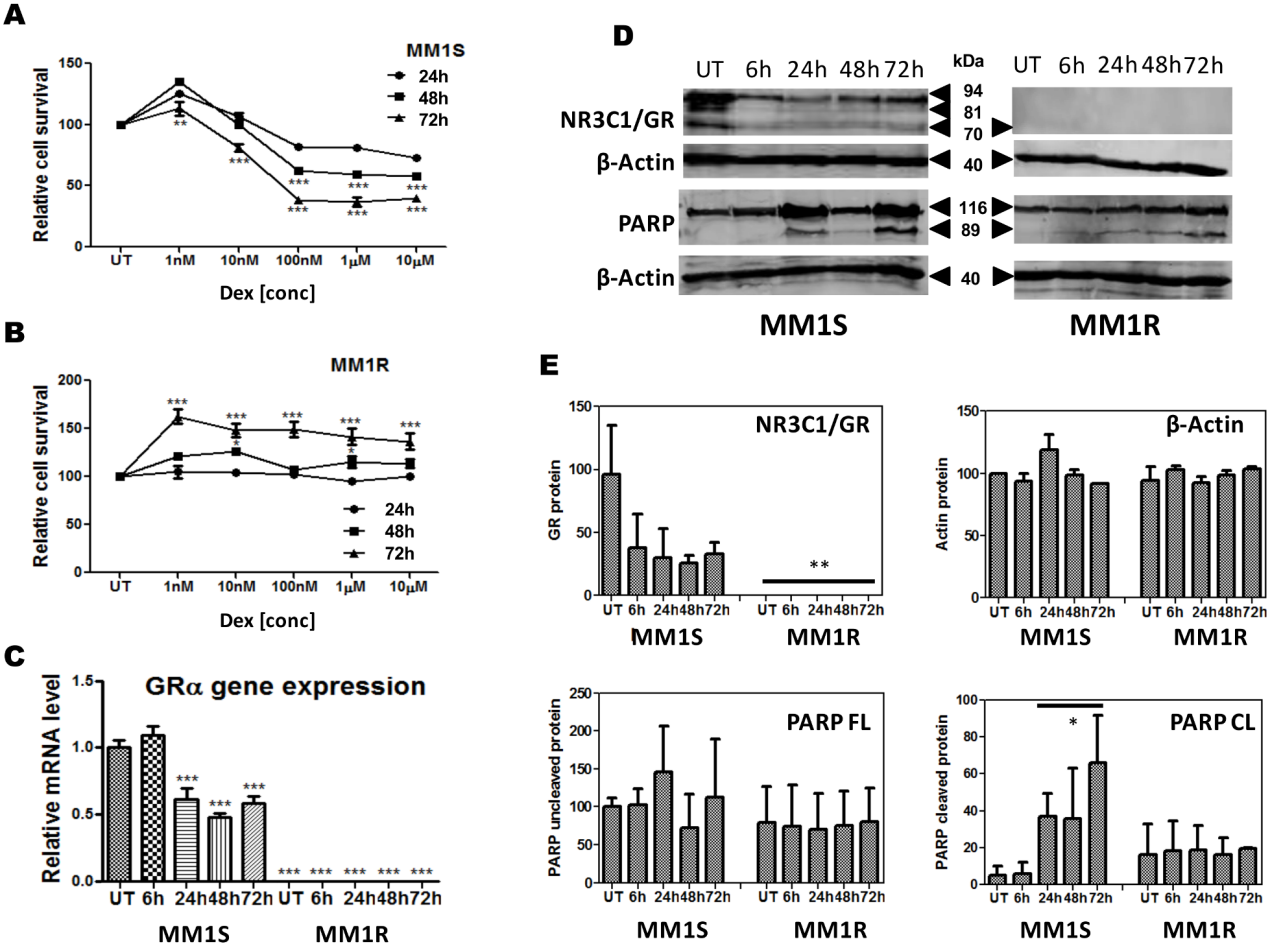
Dexamethasone decreases cell viability in a dose and time dependent manner in GC sensitive MM1S but not GC resistant MM1R cells. (**A–B**) dose response curves of MM1S and MM1R cells treated with dexamethasone at different concentrations for 24, 48 and 72 h as determined by MTT colorimetric assay. Data represent (mean ± SEM) values of three independent experiments. (**C**) time dependent changes in GRα mRNA levels upon 1 µM dexamethasone treatment. Data represent (mean ± SEM) values of three independent experiments normalized to 28sRNA housekeeping gene and relative to MM1S untreated condition (S UT). Means with ***, **, * are significantly different (p<0.001, <0.01 or <0.05 and ns are not significantly different as determined by one-way ANOVA (Dunnett’s Post-test) (**D**) Time dependent changes in GRα protein levels and PARP cleavage in MM1S and MM1R cells following treatment of 1 µM Dex. (**E**) Densitometric analysis of western blot signals (n = 2) of protein levels of GR (92 kDa), β-Actin (40 kDa), full length PARP (116 kDa) and cleaved PARP (89 kDa) in MM1S and MM1R, following treatment of 1 µM Dex. Significant differences between western signals in MM1R and MM1S are indicated.

Before investigation of the regulatory role of GC inducible microRNA in NR3C1/GR responsive gene expression and cell death responses, we first characterized the GC therapy response in MM1S and MM1R cells. Briefly, MM1S and MM1R cells were left untreated or treated with 1 µM of the synthetic GC agonist dexamethasone (Dex) for respectively 6 h, 24 h, 48 h, 72 h. mRNA and proteins were isolated at each time point and analysed for corresponding NR3C1/GRα expression. Treating the cells with different concentrations of Dex for 24 h, 48 h and 72 h followed by measuring cell viability by a MTT colorimetric assay, revealed a dose and time dependent decrease in cell survival of MM1S cells upon GC exposure (Fig. 1A & 1B). In contrast Dex lacks the capacity to induce cytotoxicity in MM1R cells and rather triggers a weak growth stimulatory effect [28], [35], [36], [37]. In line with the different GC response in both cell lines, QPCR analysis demonstrates significant NR3C1/GRα mRNA transcription in MM1S, but near background levels of NR3C1/GRα mRNA in MM1R cells (Fig. 1C). Western analysis finally confirms significant levels of GRα protein expression in MM1S and undetectable levels of GRα protein in MM1R cells (Fig. 1D–E). As can be observed from Fig. 1D, MM1S cells express multiple translational isoforms of GRα [38], [39], of which expression decreases following GC treatment.

Despite the time dependent reduction of GR mRNA and protein levels upon 24–72 h GC treatment (Fig. 1C–1D), the cell death response in MM1S is not abrogated. Furthermore, GC induced cell death triggers time dependent cleavage of the apoptotic marker PARP in MM1S but not in MM1R cells (Fig. 1D–E). In contrast to GR levels observed in MM1S, expression levels of most GR isoforms in MM1R are below detection limit, although background levels of a 70 kDa variant can still be detected upon prolonged exposure (data not shown). Remarkably, although Dex completely lacks capacity to induce cytotoxicity in MM1R cells, a weak growth stimulatory effect can be observed upon prolonged exposure of GC in MM1R cells. This maybe attributed to residual activity of a possible anti-apoptotic translational isoform of GR [38].

### Dexamethasone triggers GC specific transcriptional changes in cell cycle, DNA damage/repair, cell death and immunological pathways in MM1S but not MM1R

**Figure 2:**
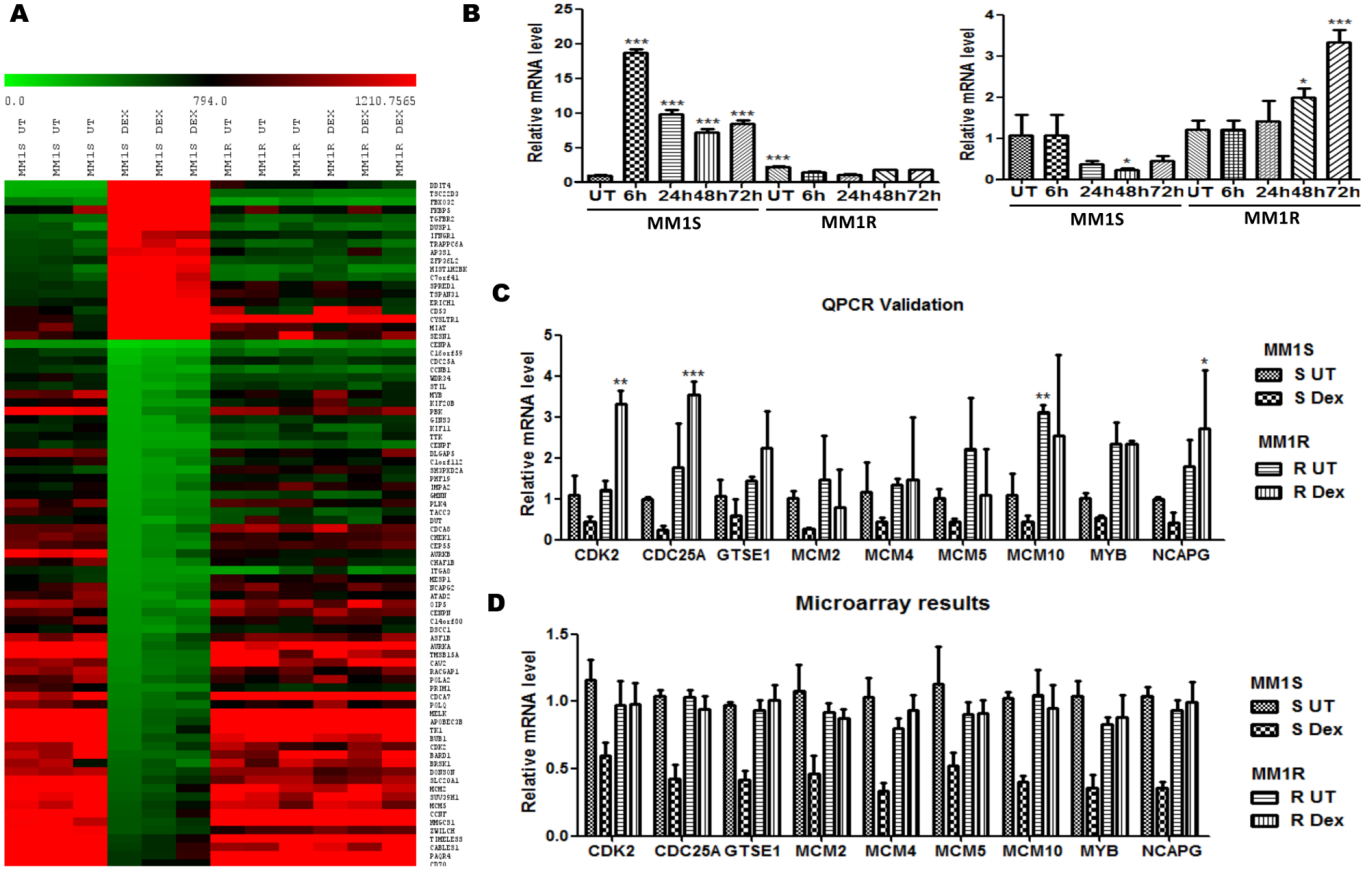
Dexamethasone triggers GC specific transcriptional changes in cell cycle, DNA damage/repair, cell death and immunological pathways in MM1S but not MM1R. (**A**) The heat map represents relative gene expression levels of MM1S and MM1R cells, left untreated (solvent) or treated with 1 µM Dex for 72 h. Gene expression changes >2-fold and with a p-value <0.05 are plotted on the heat map. (**B**) **Left panel:** Realtime quantitative PCR (QPCR) validation of Dex dependent activation of GILZ (TSC22D3) mRNA transcription **Right panel:** Realtime quantitative PCR (QPCR) validation of Dex dependent repression of CDK2 mRNA in MM1S but not in MM1R. Means with ***, **, * are significantly different (p<0.001, <0.01 or <0.05 respectively) as compared to control setups, determined by one-way (Dunnett’s Post-test) (**C–D**) Realtime quantitative PCR (QPCR) validation and Illumina microarray observation of the top GC regulated genes involved cell cycle, DNA damage/repair and cell death in S = MM1S but not R = MM1R. All the QPCR validation studies have been normalized to the 28S RNA housekeeping gene and are represented relative to MM1S untreated condition (S UT). Bar graphs represent relative mRNA (mean ± SEM) levels of three independent experiments. Means with ***, **, * are significantly different (p<0.001, <0.01 or <0.05 respectively) from control setups as determined by two-way ANOVA (Bonferroni post-tests).

To evaluate specific gene expression changes linked to GC therapy response in multiple myeloma, mRNA was isolated from three independent experiments from MM1S and MM1R cells exposed for 72 h to the GC Dex (1 µM). After mRNA isolation, genome-wide transcription profiles were determined by Illumina array hybridisation. Results were analysed with the R-package “Limma” (v3.14.1) [40], [41], which resulted in the identification of 268 downregulated and 151 upregulated genes in MM1S, after filtering for a minimal two-fold transcriptional change in Dex treated samples versus control setup (absolute fold change >2) and adjusted P-value <0.05 (Gene Expression Omnibus (GEO) database GSE59805). In MM1R, no significant Dex responsive changes could be detected, in line with the GC resistant phenotype and the absence of GRα in MM1R cells (see Fig. 1). The heatmap (Fig. 2A) represents a graphical presentation of Dex specific transcriptional changes in MM1S and lack of Dex response in MM1R cells in three biological replicates, revealing highly reproducible and robust patterns of gene expression. By QPCR analysis, we confirmed GC dependent transactivation of GILZ (TSC22D3) target gene as well as GC specific (trans)repression of CDK2 in MM1S but not in MM1R, in line with the Illumina array data and previous reports [37], [42], [43], [44] (Fig. 2B). Remarkably, prolonged GC exposure (72 h) of MM1R cells reveals weakly increased levels of CDK2 according to QPCR analysis, which could not be detected by less sensitive microarray analysis. This effect may be attributed to residual activity of anti-apoptotic GR isoforms in MM1R, which may also contribute to weak growth stimulatory effects (Fig. 1A) upon prolonged exposure [38]. Integrated pathway analysis (IPA) further revealed that GCs trigger gene expression changes which promote cell death, decrease cell proliferation (cell cycle, DNA replication) and regulate immunological haematological pathways in MM1S but not MM1R (Table 1), in line with the reduced cell viability assay in MM1S (see Fig. 1). Next, we further validated microarray results of some additional target genes involved in cell cycle, DNA damage/repair and cell death. As can be observed from (Fig. 2C & 2D), the QPCR validation results correlate with the obtained microarray data. Furthermore, by analyzing patterns of coregulated genes, IPA software allows to identify activation or inhibition of possible upstream regulators (kinases, receptors, transcription factors) involved in gene expression changes [45]. The latter analysis predicts highly significant activation of Dex liganded NR3C1 and TP53 transcription factors, in regulating MM1S cell cycle arrest and/or apoptosis [46] (Table 2). Furthermore, predicted activation of IKK and cyclin dependent kinases CDKN2A, CDKN1A may be indicators of DNA damage/cell death and cell cycle events triggered by Dex [47], [48], [49], [50]. Finally, possible inhibition of anti-apoptotic prosurvival signals stimulated by TNF and AKT kinases may also contribute to Dex induced cell death in MM1S (Table 2).

**Table 1:** Top molecular and cellular biofunctions upon 1 µM Dex treatment in MM1S cells for 72 h.

Cellular function	P-value	Predicted Activating state	Z-score
Cell Death and Survival	1.03E-12	Increased	3.301
Cellular Assembly and Organization,DNA Replication, Recombination, Repair	1.98E-09	Decreased	−2.711
Cell Cycle	9.63E-09	Decreased	−2.043
DNA Replication, Recombination, and Repair	2.89E-07	Decreased	−2.22
Cellular Assembly and Organization,DNA Replication, Recombination, Repair	9.74E-07	Decreased	−2.138
Cellular Development, CellularGrowth and Proliferation	1.28E-06	Decreased	−3.067
Cellular Growth and Proliferation	1.81E-06	Decreased	−3.185
Cell Cycle	5.96E-06	Decreased	−2.205
Cell Cycle	7.98E-06	Decreased	−2.305
Cancer, Hematological Disease,Immunological Disease	1.04E-05		
Immunological Disease	2.27E-05		

**Table 2:** Top upstream regulators upon 1 µM Dex treatment in MM1S cells for 72 h.

Upstreamregulator	Molecule type	P-value	Predicted activation state	Activation Z-score
TP53	Transcription regulator	1.53E-49	Activated	5.504
CDKN1A	Kinase	5.31E-47	Activated	4.322
CDKN2A	Transcription Regulator	1.80E-37	Activated	6.844
Dexamethasone	Chemical drug	1.14E-11	Activated	2.719
NR3C1	Ligand-dependent nuclear receptor	4.58E-08	Activated	2.359
I kappa b kinase	Complex	4.09E-07	Activated	2.629
PPARG	ligand-dependent nuclear receptor	1.35E-05	Inhibited	−2.804

### MicroRNAs responsive to GC in MM1S cells, but unresponsive to GC in MM1R cells, are predicted to modulate cell cycle, cell growth and proliferation pathways

**Figure 3:**
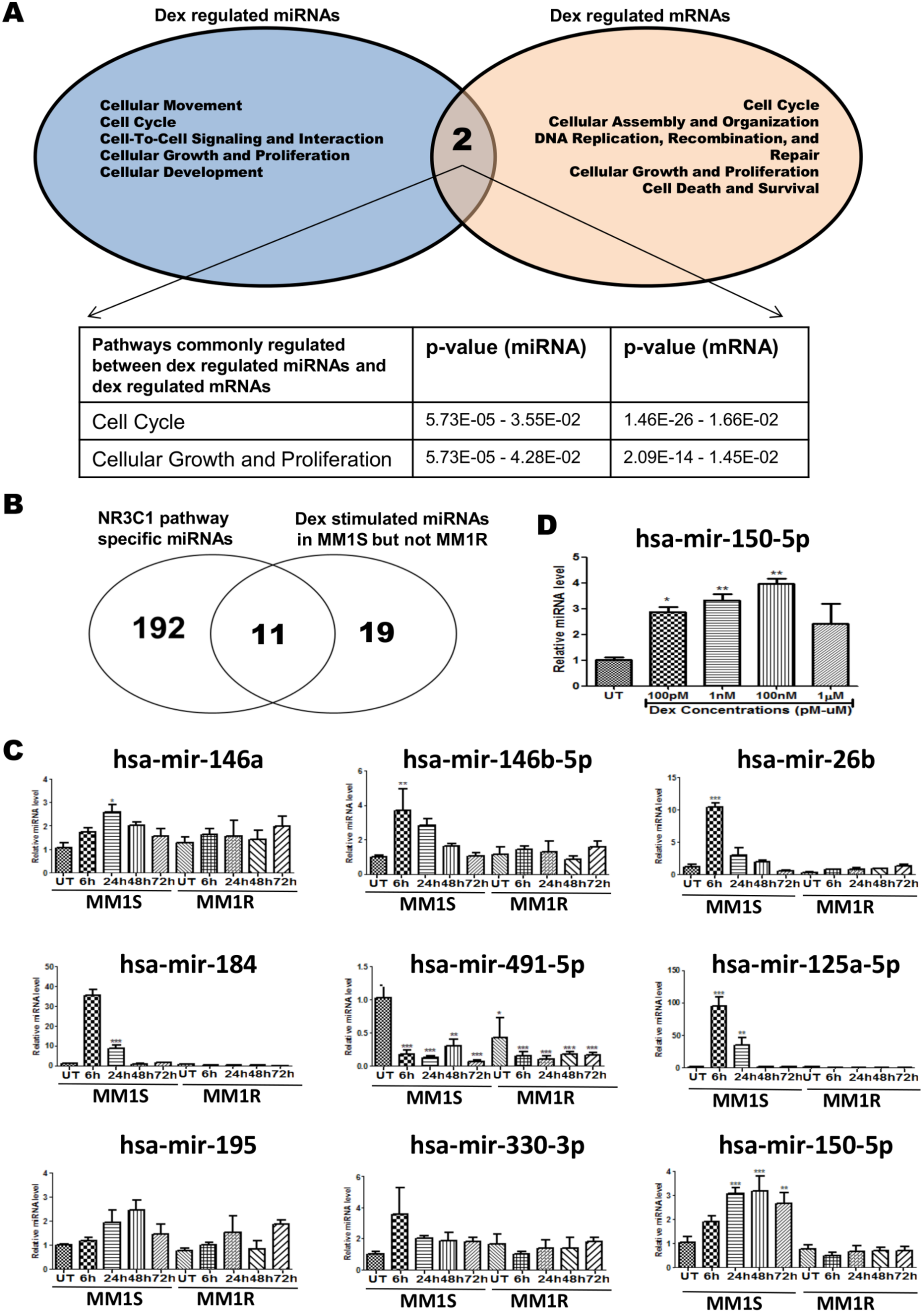
Experimental validation of GC inducible microRNAs, potentially involved in regulation of the NR3C1(GR) pathway in MM1S cells (**A**) Venn-diagram which summarizes the overlap between the top 5 predicted molecular and cellular functions generated by IPA from GC regulated miRNAs and GC regulated mRNAs (**B**) Venn-diagram which represents the crosscomparison of the list of GC responsive miRNAs identified in MM1S with the list of GR(NR3C1) pathway specific miRNAs according to MiRWalk prediction freeware. (**C**) Real-time QPCR validation of the transcription levels of 9 common miRNAs selected in Fig. 3B, in MM1S and MM1R cells treated for 24, 48 or 72 h with 1 µM Dex. (**D**) GC dose responsive regulation of mir-150-5p levels in MM1S cells treated for 72 h with GC. All the miRNA QPCR validation studies have been normalized to SNORD 95 and SNORD 96A housekeeping miRNAs according to manufacturer instructions and relative to MM1S untreated condition (S UT). Bar graphs represent relative miRNA (mean ± SEM) levels of three independent experiments as compared to control setups. Means with ***, **, * are significantly different to control setups (p<0.001, <0.01 or <0.05) as determined by one-way ANOVA (Dunnetts Post test).

Total RNA was isolated from MM1S and MM1R cells treated with 1 µM Dex for 72 h, qPCR profiling of 760 miRNAs as previously described [32]. This allowed to identify 30 GC inducible and 14 GC repressed miRNAs (>2-fold) in MM1S, which do not change transcription in MM1R cells (summarized in Table 3). Next, IPA analysis was applied to cross compare microRNA and mRNA profiles in MM1S to predict target genes and top pathways which are affected by microRNA expression. Venn diagram analysis of the top 5 ranked molecular and cellular pathways enriched for genes differentially expressed in MM1S upon Dex exposure and predicted microRNA target genes responsive to Dex treatment reveals that GC responsive microRNAs may target mRNAs involved in cell cycle and cell proliferation pathways (Fig. 3A). For example, Dex inducible microRNAs mir-150-5p, mir-152, mir-146b-5p, mir-1290, mir-125a-5p, mir-1206 are predicted to modulate multiple target genes involved in cell cycle, cellular growth and proliferation pathways (as yellow marked in Table S1).

**Table 3:** Dex regulated miRNAs in MM1S but not MM1R upon 1 µM Dex treatment for 72 h.

hsa-miR	Fold activation	Log2FC	hsa-miR	Fold repression	Log2FC
hsa-miR-146a	2.10E+00	1.4	hsa-miR-492	3.62E+08	0.00
hsa-miR-152	2.17E+00	1.5	hsa-miR-1275	4.13E+04	0.00
hsa-miR-491-5p	2.18E+00	1.5	hsa-miR-766	3.14E+04	0.01
hsa-miR-28-5p	2.20E+00	1.5	hsa-miR-412	6.25E+03	0.01
hsa-miR-590-5p	2.20E+00	1.5	hsa-miR-636	3.81E+03	0.02
hsa-miR-454#	2.23E+00	1.5	hsa-miR-181c	3.34E+03	0.02
hsa-miR-146b-5p	2.24E+00	1.5	rno-miR-29c#	2.70E+03	0.02
hsa-miR-627	2.43E+00	1.6	hsa-miR-603	1.91E+03	0.02
hsa-miR-26b	2.58E+00	1.6	hsa-miR-100#	1.64E+03	0.02
hsa-miR-95	2.69E+00	1.6	hsa-miR-17#	3.71E+02	0.05
hsa-miR-324-3p	3.16E+00	1.8	hsa-miR-106b#	1.03E+01	0.31
hsa-miR-150-5p	3.54E+00	1.9	hsa-miR-27b	5.46E+00	0.43
hsa-miR-484	3.91E+00	2.0	hsa-miR-92a-1#	4.61E+00	0.47
hsa-miR-1290	4.07E+00	2.0	hsa-miR-301b	2.11E+00	0.69
hsa-miR-652	4.09E+00	2.0			
hsa-miR-330-3p	4.19E+00	2.0			
hsa-miR-195	4.62E+00	2.1			
hsa-miR-184	8.25E+00	2.9			
hsa-miR-125a-5p	2.49E+02	15.8			
hsa-miR-126	2.67E+02	16.4			
hsa-miR-515-3p	3.28E+02	18.1			
hsa-miR-623	4.98E+02	22.3			
hsa-miR-1206	8.74E+02	29.6			
hsa-miR-802	1.01E+03	31.7			
hsa-miR-451	1.11E+03	33.4			
hsa-miR-892b	4.12E+03	64.2			
hsa-miR-629	4.78E+03	69.1			
hsa-miR-518b	7.14E+03	84.5			
hsa-miR-663B	4.05E+04	201.3			
hsa-miR-34B	5.69E+05	754.2			

**Figure 4:**
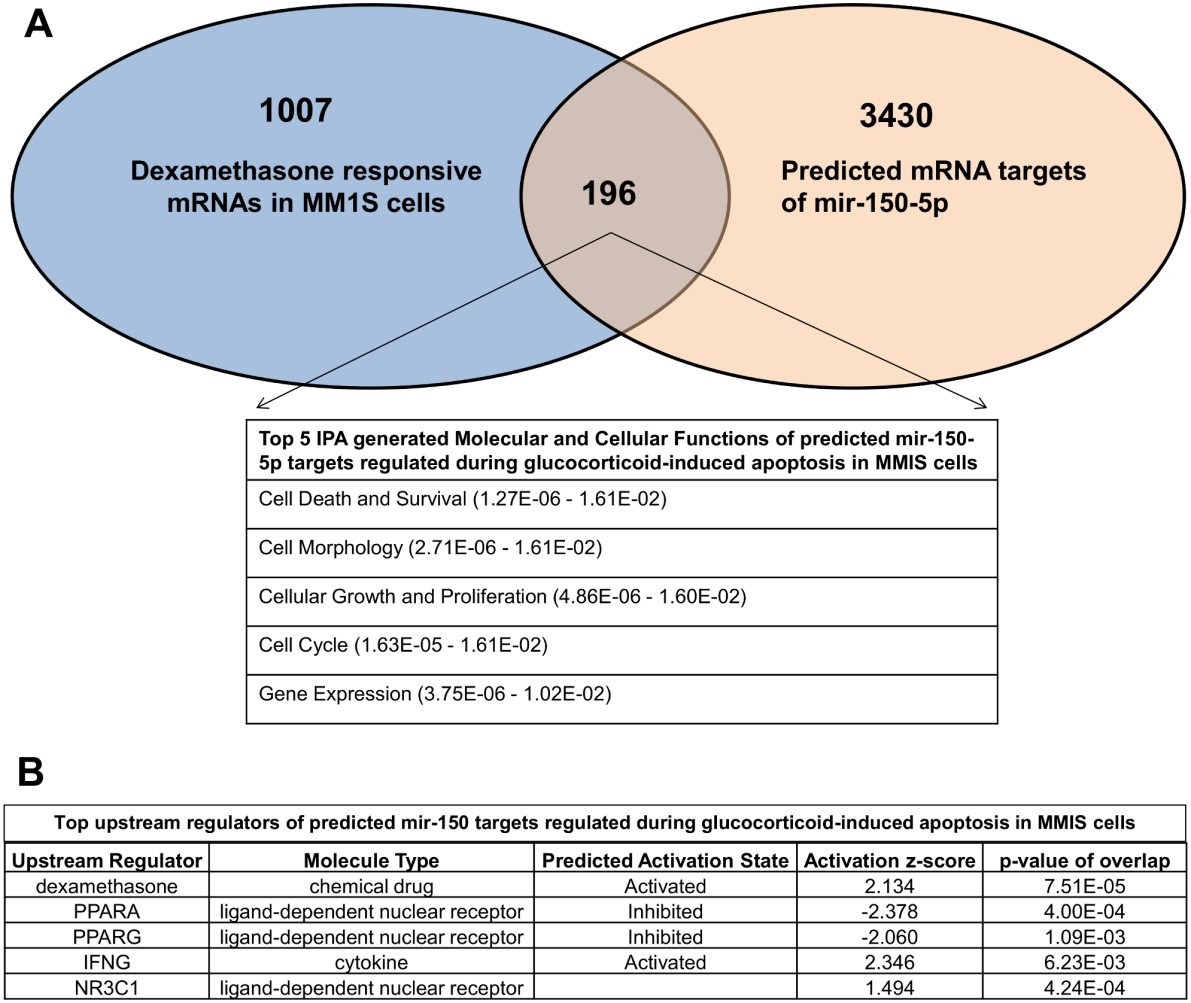
Predicted role for mir-150-5p in regulation of cell death and survival, cell cycle, cellular growth and proliferation in MM1S cells. (**A**) Venn-diagram which represents the crosscomparison of 3626 predicted hsa-mir-150-5p targets according to miRWalk software and 1203 dexamethasone responsive target genes in MM1S cells, which remain unaffected in MM1R cells (**B**) IPA upstream analysis of the predicted microRNA target mRNAs in MM1S cells.

To reduce the number of Dex responsive microRNA for further validation and functional studies, we next cross compared the list of GC responsive miRNA identified in MM1S with a list of miRNAs predicted to regulate the GR(NR3C1) pathway (Biocarta) according to the MiRWalk database [34] (Fig. 3B). This generated a shortlist of 11 miRNAs, which were selected for further QPCR validation. Along the same line, IPA correlation of Dex repressed mRNAs and 30 Dex inducible miRNAs identified a similar GR specific role for mir-150-5p, mir-146b-5p and mir-125a-5p in the cell cycle and cellular growth and proliferation pathway (Table S1). Time dependent GC responsive changes in transcription levels of 11 selected microRNAs were evaluated by QPCR in MM1S and MM1R cells exposed for different time points (6 h, 24 h, 48 h and 72 h) to 1 µM of Dex. QPCR analysis could confirm time and GC specific induction of 5 hsa-microRNAs i.e. of mir-26b, mir-125a-5p, mir-146-5p, mir-150-5p, and mir-184 (Fig. 3C). Finally, hsa-mir-150-5p was selected for further functional studies because of its persistent GC responsive transcription between 6 and 72 h, and GC dose dependent regulation of mir-150-5p following 72 h treatment (Fig. 3D). Upon cross comparing 1203 Dex responsive target genes from MM1S cells with 1 µM Dex treatment for 72 h (gene selection was done based on p-value <0.05 and absolute fold change >1.5) and 3626 predicted hsa-mir-150-5p targets via the miRWalk database (prediction by at least 4 out of 10 available algorithms, i.e. DIANAmT, miRanda, miRDB, miRWalk, RNAhybrid, PICTAR4, PICTAR5, PITA, RNA22 and Targetscan), 196 common mRNA/gene targets (Fig. 4A) were identified in MM1S, which remain unaffected in MM1R cells (detailed list of genes can be found in Table S2). Upon employing IPA analysis, the 196 mRNA predicted GC responsive mir-150-5p target genes are enriched for cell death, cell morphology, cell cycle, cellular growth and proliferation pathways (Fig. 4A). Of special note, IPA upstream analysis predicts that mir-150-5p gene target genes are responsive for regulation by GC (Dex)/NR3C1, PPAR or STAT/IFNγ signaling (Fig. 4B).

### Transfection of a mir-150-5p mimetic triggers GR specific gene expression in MM1S but not in MM1R cells, without modulating GR protein levels

**Figure 5:**
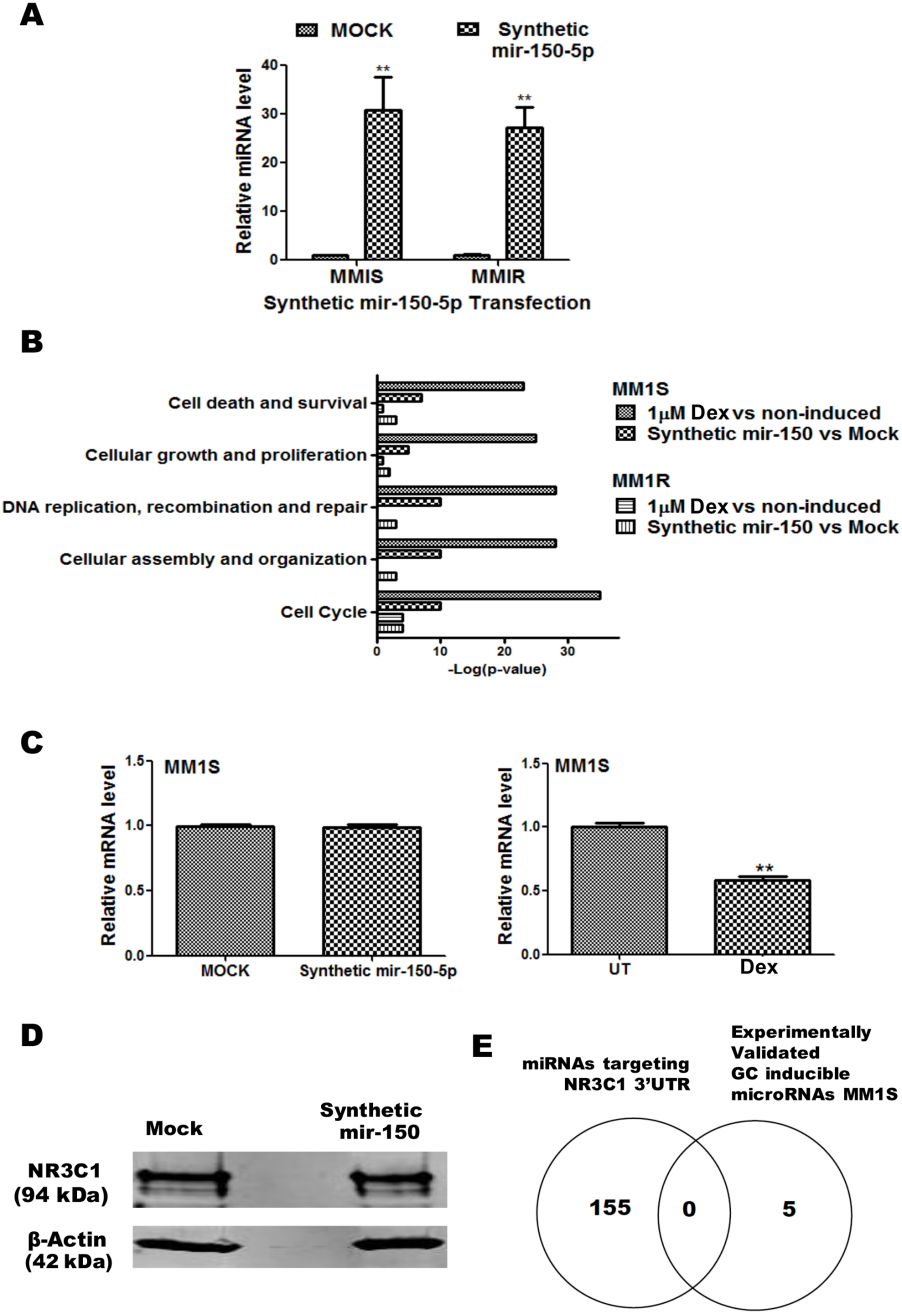
Mir-150-5p mimetic transfection triggers GR specific gene expression in MM1S but not in MM1R cells, without modulating GR protein levels. (**A**) QPCR based quantification of synthetic mir-150-5p levels present 72 h after transfection of MM1S and MM1R cells. The miRNA expression levels have been normalized to SNORD 95 and SNORD 96A housekeeping miRNAs and are presented relative to MOCK control. Bar graphs represent relative miRNA (mean ± SEM) levels of three independent experiments. Means with ***, **, * are significantly different (p<0.001, <0.01 or <0.05) from control setups according to two-way ANOVA (Bonferroni post-tests). (**B**) Comparison of top enriched pathways (IPA) of mRNA changes in MM1S cells treated for 72 h to 1 µM Dex versus changes in MM1S and MM1R cells after 72 h mock transfection of transfection with synthetic mir-150-5p. (**C**) NR3C1/GRα protein levels detected following 72 h transfection of a synthetic mir-150-5p mimetic in MM1S cells. (**D**) Venn-diagram which represents the overlap of experimentally validated GC inducible microRNAs in MM1S cells with the list of miRNAs predicted to target the 3′UTR of NR3C1. (**E**) QPCR analysis of NR3C1/GRα mRNA levels present in MM1S cells following 72 h transfection of a synthetic mir-150-5p mimetic (left panel) or following 72 h Dex (1 µM) treatment (right panel).

To validate possible mir-150-5p effects on the predicted mRNA targets and/or cell cycle or cell proliferation pathways, we performed transfection experiments in MM1S and MM1R cells with a synthetic mir-150-5p mimetic in comparison to Dex treatment. Remarkably, although MM1S and MM1R show similar transcription levels of mir-150-5p upon mimetic transfection (Fig. 5A), we could only observe significant changes in mir-150-5p responsive mRNAs in MM1S but not in MM1R cells, upon analysing Illumina mRNA profiles. Interestingly, upon cross comparing Dex treated and mir-150-5p mimetic transfected MM1S/MM1R cells, IPA analysis revealed a remarkable overlap in mRNAs affected related to cell cycle-proliferation, DNA repair and cell death pathways in MM1S, as well as lack of effects for both setups in MM1R cells (Fig. 5B) (Table 4 and 5). To evaluate whether mir-150-5p may change GR/NR3C1 mRNA levels or protein stability, we performed QPCR and Western blot analysis following mir-150-5p transfection in MM1S cells. From Fig. 5C and D, it can be observed that mir-150-5p transfection does not change GR/NR3C1 mRNA or protein levels respectively. These results are in line with the in silico data, showing that none of the validated GC-inducible miRs are targeting GR-3′UTR mRNA (Fig. 5E and Table S3). Altogether, these results suggest that the mir-150-5p may mediate indirect GR like effects.

**Table 4:** Top molecular and cellular functions regulated upon synthetic mir-150-5p transfection in MM1S cells.

Category	p-Value	Predicted Activation State	Activation z-score
Cell Cycle	3.11E-06	Decreased	–2.232
Cellular Growth and Proliferation	7.90E-05	Decreased	–2.94
Cellular Assembly and Organization	2.45E-04	Decreased	–2
DNA Replication, Recombination,and Repair	2.45E-04	Decreased	–2
Cellular Movement	9.24E-03	Decreased	–2.201
Cell-To-Cell Signaling and Interaction	9.24E-03	Decreased	–2.201
Immune Cell Trafficking	9.24E-03	Decreased	–2.201
Inflammatory Response	9.24E-03	Decreased	–2.201

**Table 5:** Top upstream regulators in mir-150-5p transfected MM1S cells.

UpstreamRegulator	FoldChange	MoleculeType	Predicted Activation State	Activation z-score	p-value ofoverlap
TP53	1.156	transcription regulator	Activated	2.056	9.11E-23
CDKN1A	–1.86	kinase	Activated	2.433	1.91E-13
IL6	–1.027	cytokine	Inhibited	–2.407	3.40E-13
CDKN2A	1.08	transcription regulator	Activated	2.656	1.44E-09
dexamethasone		chemical drug	Activated	2.668	3.35E-09
NR3C1	–1.218	ligand-dependent nuclear receptor	Activated	2.007	1.07E-05
PPARG	1.04	ligand-dependent nuclear receptor	Inhibited	–3.153	5.40E-05
NFkB (complex)		complex	Inhibited	–2.669	3.71E-03

**Figure 6:**
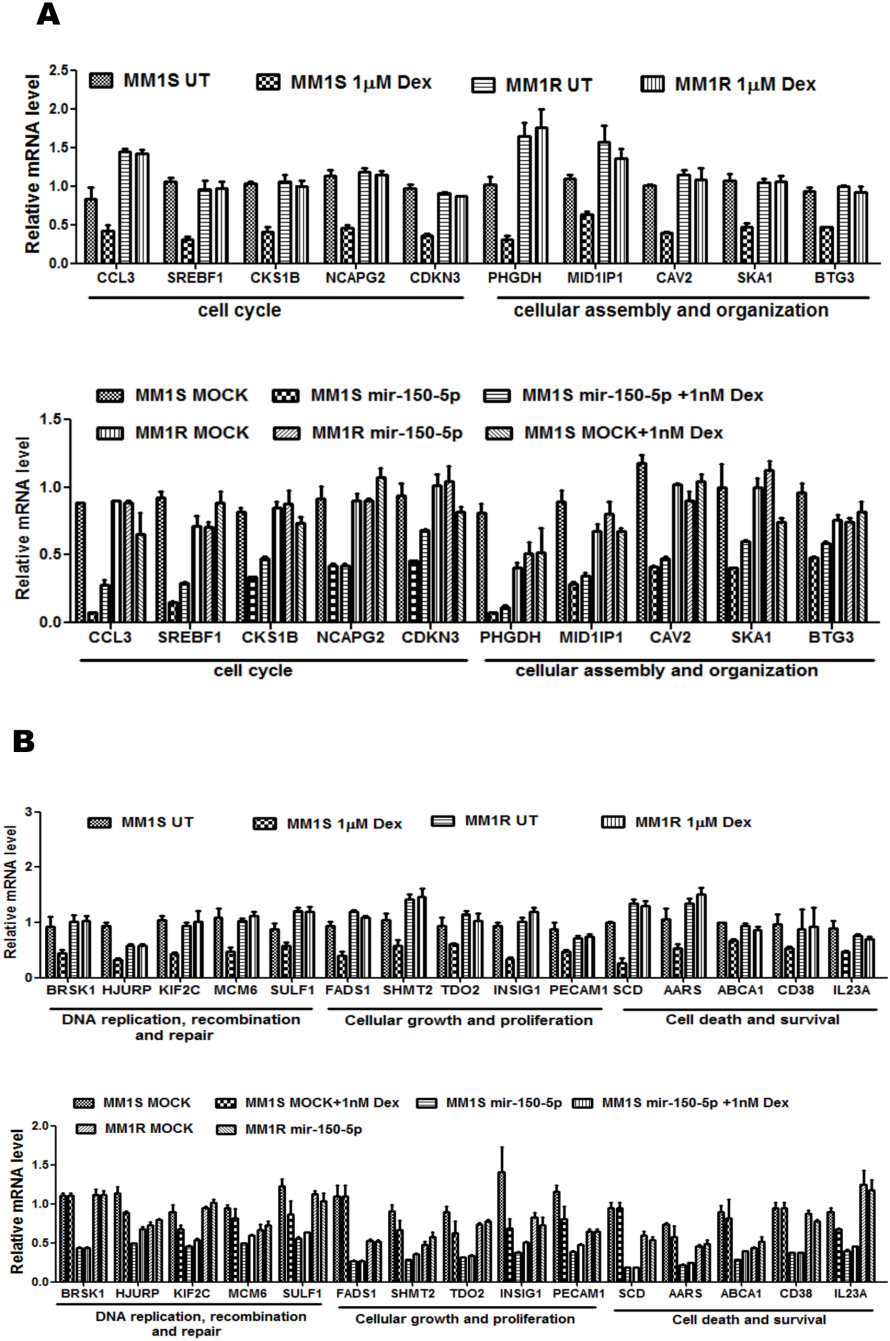
Mir-150-5p specific mRNA changes in MM1S are associated with pathways related to cell death and survival, cell cycle, cellular growth and proliferation (A–B) mir-150-5p specific mRNA regulation of the genes involved in (i) cell cycle, (ii) cellular assembly and organization, (iii) DNA replication, recombination and repair, (iv) cell growth and proliferation, (v) cell death and survival. Illumina BeadChip Gene Expression Array results are presented as bar graphs, reflecting mean of gene expression fold change from three independent experiments of MM1S cells, treated for 72 h with 1 µM Dex vs control cells (upper panels A–B), or else of MM1S cells transfected for 72 h with synthetic mir-150-5p versus mock transfection, treated for 72 h with 1 nM Dex or a combination thereof versus control samples (lower panels A–B).

**Figure 7:**
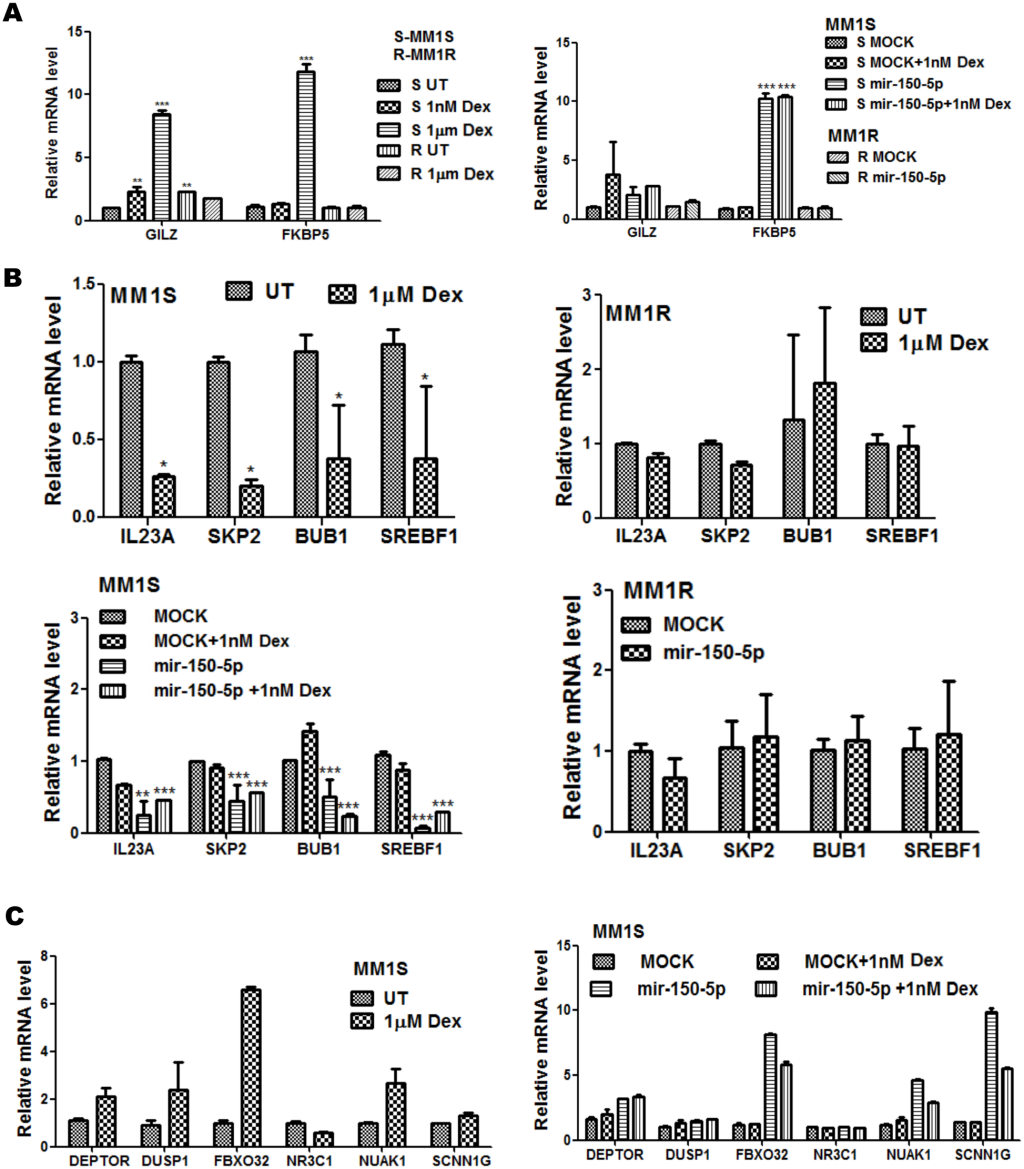
Mir-150-5p transfection mimics GR like gene activation and repression response in MM1S and lack of response in MM1R cells. (**A**) Realtime quantitative PCR (QPCR) validation of GILZ (TSC22D3) and FKBP5 mRNA induction in MM1S and in MM1R cells, following 72 h treatment with 1 nM or 1 µM Dex (left panel), or following 72 h transfection with synthetic mir-150-5p, 72 h treatment with 1 nM Dex, or a combination thereof (right panel). Data represent (mean ± SEM) values of three independent experiments normalized to 28S RNA housekeeping gene and relative to the respective untreated/solvent (UT) or MOCK control setups. Means with ***, **, * are significantly different (p<0.001, <0.01 or <0.05) from control setup as determined by two-way ANOVA (Bonferroni posttest). (**B**) Realtime quantitative PCR (QPCR) validation of MYB, IL23A, SKP2, BUB1, SREBF1 mRNA repression levels in MM1S or MM1R cells, following 72 h treatment with 1 nM or 1 µM Dex, or following 72 h transfection with synthetic mir-150-5p, 72 h treatment with 1 nM Dex, or a combination thereof. Data represent (mean ± SEM) values of three independent experiments normalized to 28S RNA housekeeping gene and relative to the respective untreated/solvent (UT) or MOCK control setups. Means with ***, **, * are significantly different (p<0.001, <0.01 or <0.05) from control setup as determined by two-way ANOVA (Bonferroni posttests) (**C**) Illumina BeadChip Gene Expression Array results of selected genes are presented as bargraphs, reflecting mean of gene expression fold change from three independent experiments of MM1S cells, treated for 72 h with 1 µM Dex, or else of MM1S cells transfected for 72 h with synthetic mir-150-5p versus mock transfection, treated for 72 h with 1 nM Dex or a combination thereof versus control samples.

The genes involved in cell cycle-proliferation, DNA repair and cell death pathways which are regulated in Dex treated and mir-150-5p mimetic transfected MM1S/MM1R cells are indicated in Figure 6A and 6B or summarized in Table S4. To further characterize crosstalk between GC treatment and mir-150-5p transfection, we also performed microarray analysis of combination setups of mir-150-5p with 1 nM Dex, since 1 µM Dex treatment combinations did not yield array quality RNA because of excessive cytotoxicity. As can be noted from Fig. 6A and 6B, 1 nM Dex treatment triggers weaker gene expression changes as compared to 1 µM Dex treatment. Furthermore, since mir-150-5p levels upon transfection increase 30-fold (Fig. 6A) as compared to 3-fold induction upon Dex treatment (Fig. 3D), mir-150-5p mimetic overexpression triggers stronger gene expression changes than 1 nM Dex treatment. As such, combining mir-150-5p transfection with 1 nM Dex treatment did not further increase gene expression changes elicited by mir-150-p transfection alone (Fig. 7A & B). A further detailed comparison of GC and mir-150-5p responsive activation (i.e. GILZ and FKBP5) (Fig. 7A) or repression (IL23A, SKP2, BUB, SREBF1) (Fig. 7B) of typical GR target genes revealed similar effects in MM1S but lack of effects in MM1R. For example, transactivation of the FKBP5 and GILZ genes can be observed following mir-150-5p transfection and GC treatment in MM1S cells, although mir-150-5p effects on FKBP5 expression are stronger. Along the same line, repression of GR target genes IL23A, SKP2, BUB or SREBF1 can be observed upon Dex treatment as well following mir-150-5p transfection. Finally, besides the common regulated genes, additional GR target genes could be identified which were more selectively affected by mir-150-5p transfection (SCNN1G) or else GC treatment (GILZ, DUSP1, SGK1) indicating sophisticated fine tuning of GR regulation (Fig. 7C).

### The GR-like gene response triggered by mir150-5p transfection is associated with complex regulation of transcription factors, hsp chaperones, kinases, cell cycle proteins, unfolded protein stress (UPR), AMPK/mTOR and chemokine specific signalling networks

**Figure 8:**
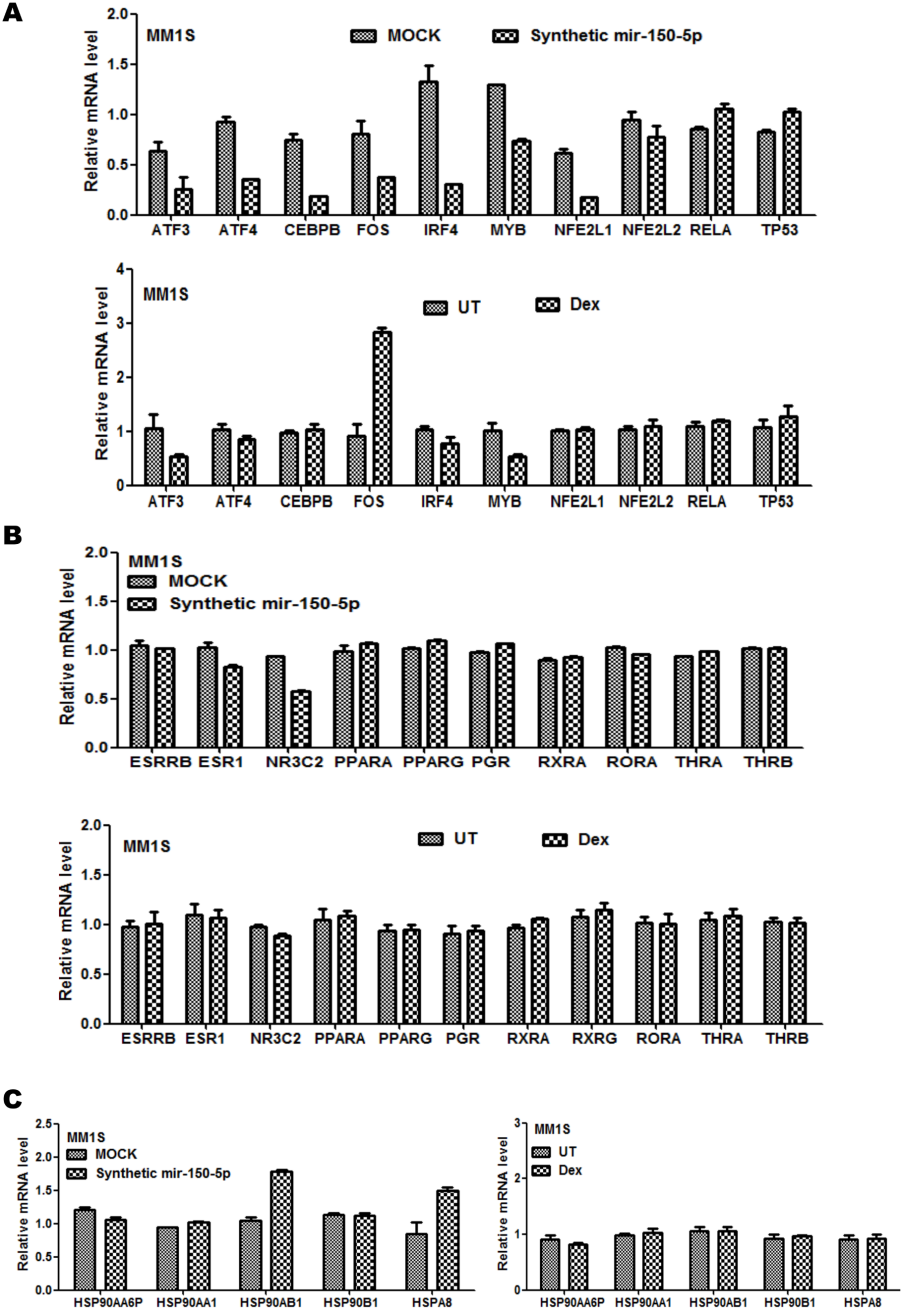
Mir-150-5p transfection is associated with complex regulation of transcription factors, hormone receptors and chaperones. Illumina BeadChip Gene Expression results of transcription factors (**A**), nuclear hormone receptors (**B**), and chaperones (**C**), are presented as bargraphs, reflecting mean of gene expression fold change from three independent experiments of MM1S, treated for 72 h with 1 µM Dex versus control setup (UT), or else of MM1S cells transfected for 72 h with synthetic mir-150-5p versus mock transfection.

Due to lack of direct mir-150-5p effects on GR(NR3C1) mRNA and protein levels, we assumed that GR like downstream effects may be caused by mir-150-5p regulation of possible GR interaction partners (transcription factors, chaperones) or alternative nuclear hormone receptors (Fig. 8A–B). Although mir-150-5p and Dex setups both decrease MYB and ATF3 mRNA levels, opposite responses were observed for FOS transcription factors. Of particular interest, MYB is a key regulator of GR dependent gene expression in leukemia [51], [52], [53], [54], [55], [56], [57] (Fig. 8A).

With respect to hormone receptors, most hormone receptors remained constant upon mir-150-5p transfection (Fig. 8B), although weakly decreased levels of NR3C2 (MR) could be detected in the setup with mir-150-5p, whereas NR3C2 levels remain stable in presence of Dex. Interestingly, as a consequence of reciprocal crosstalk of NR3C2(MR) and NR3C1(GR) actions, suppressing MR function may indeed result in ligand independent stimulation of GR actions [58]. Next, we evaluated changes in transcription levels of hormone receptor chaperone molecules upon presence of mir-150-5p. Although most HSP mRNAs remain unaffected, significant changes in HSPA8 and HSP90AB1 levels could be detected following mir-150-5p transfection but not with Dex treatment, which may further contribute in fine tuning basal activity of nuclear hormone receptors (Fig. 8C) [59], [60], [61]. Furthermore mir-150-5p selectively decreases transcript levels of ATF4, JUN, NFE2L1(NRF1), CEBP/B and IRF4 but not of NFE2L2, TP53 and RelA (Fig. 8A).

**Figure 9:**
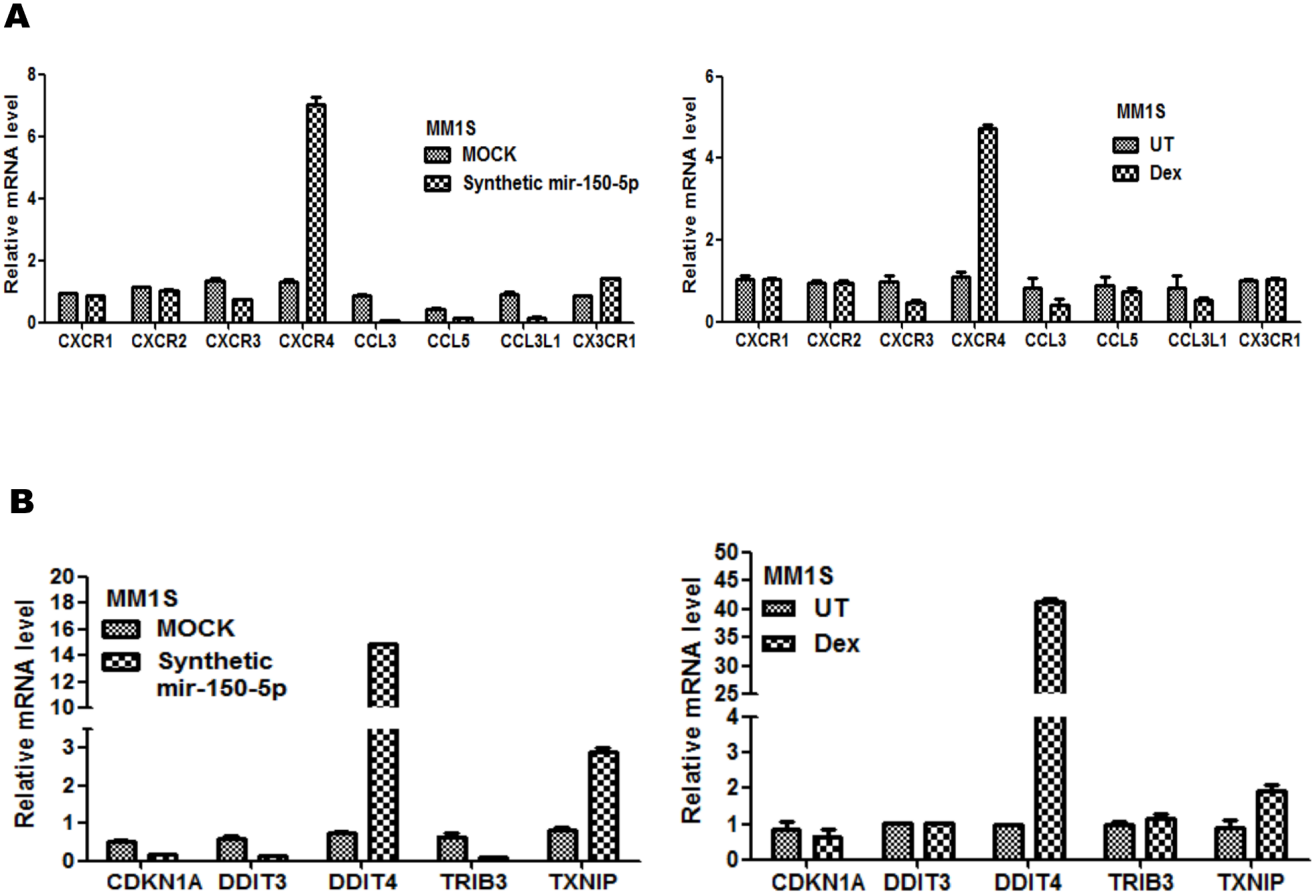
Mir-150-5p transfection is associated with complex regulation of chemokine, cell cycle, UPR/mTOR signaling. Illumina BeadChip Gene Expression results of chemokine receptors or ligands (**A**), or regulators of cell cycle, unfolded protein stress (UPR/mTOR signaling) (**B**), are presented as bargraphs, reflecting mean of gene expression fold change from three independent experiments of MM1S, treated for 72 h with 1 µM Dex versus control setup (UT), or else of MM1S cells transfected for 72 h with synthetic mir-150-5p versus mock transfection.

Finally, we also explored possible upstream effects of mir-150-5p on receptor kinase signalling pathways. Ligand independent hormone receptor (in)activation via kinases, chemokines and cytokines is well documented nowadays [62], [63], [64], [65], [66]. With respect to chemokine signalling, mir-150-5p transfection and GC treatment were found to increase mRNA levels of CXCR4 and CX3CR1 and decrease levels of CXCR3, CCL3, CCL5 whereas other members remain unaffected (Fig. 9A). Interestingly, various reports demonstrate a positive role for CXCR4/CXCL12 in hormone independent receptor activation [65], [66], [67], [68], [69]. Surprisingly, we observed a significant increase in CXCR4 levels, upon mir-150-5p transfection, in contrast to earlier reports demonstrating CXCR4 mRNA silencing of its 3′ UTR via mir-150-5p [68], [70]. Although various studies illustrate tumor promoting and prometastatic effects of CXCR4 [71], [72], its expression also plays a pivotal role in haematopoiesis [73] and has recently been reported as a good prognostic indicator in multiple myeloma [74]. Finally, we also identified remarkable effects on effectors in cell cycle (CDKN1A) and UPR/mTOR signalling pathways (DDIT3, DDIT4, TXNIP) and the Tribble family Ser/Thr pseudokinase (TRIB3) [75] which altogether may crosstalk with glucocorticoid receptor signalling and GC therapy response in MM cells [76], [77], [78] (Fig. 9B). Of special note, involvement of C/EBP and ATF4 (Fig. 8A) has been demonstrated in transcriptional regulation of UPR stress proteins DDIT3, DDIT4, TXNIP [79], [80], further corroborating our results.

### Weak sensitisation of low dose GC therapy response by mir-150-5p mimetic transfection can be reversed by transfection of a mir-150-5p-specific antagomir in MM1S cells

**Figure 10:**
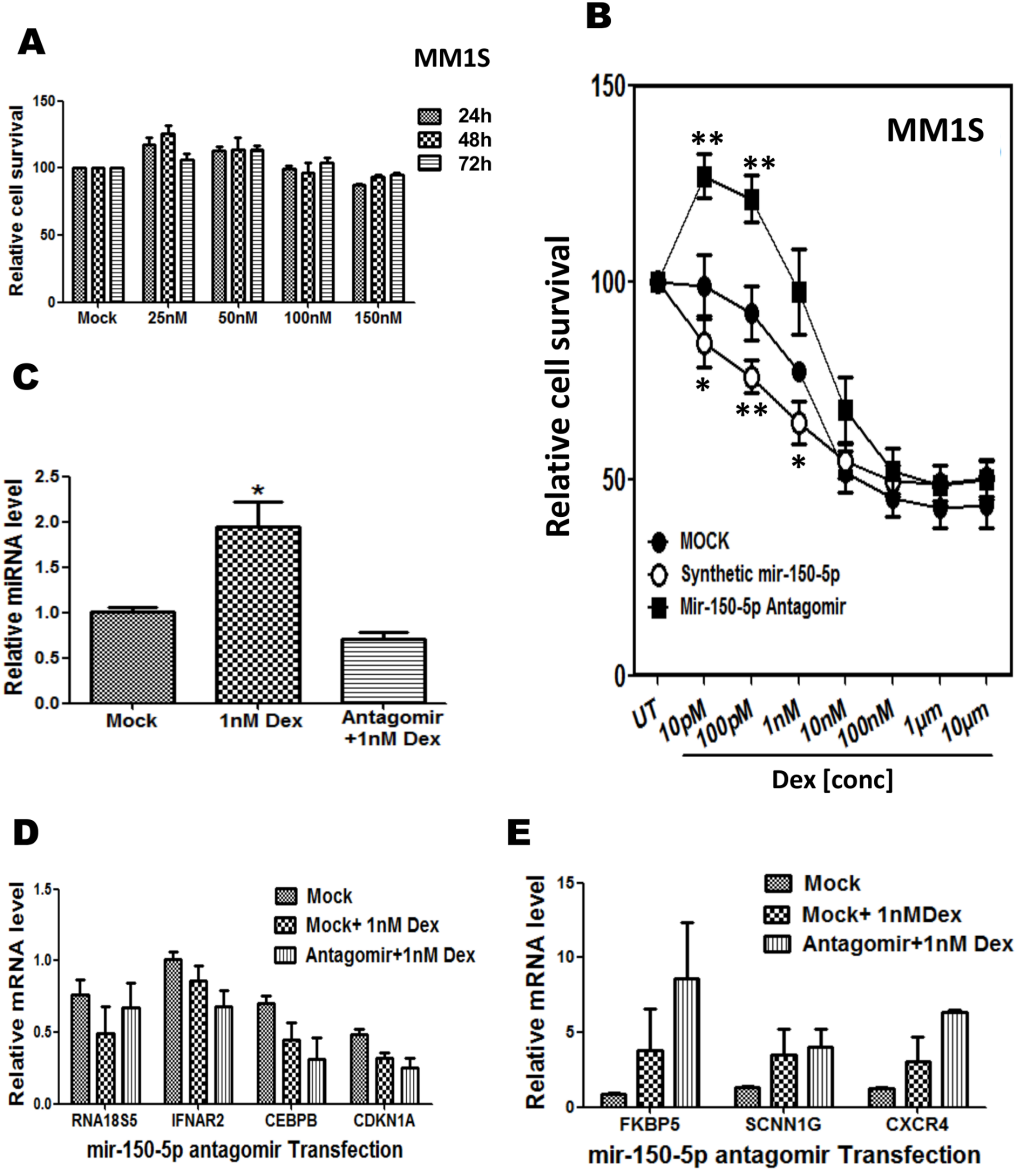
Weak sensitisation of low dose GC therapy response by mir-150-5p mimetic transfection can be reversed by transfection of a mir-150-5p-specific antagomir in MM1S cells. (**A**) MTT based quantification of MM1S cell survival, following 24, 48, or 72 h transfection with increasing doses of synthetic mir-150-5p mimetic as compared to mock transfection control (**B**) MTT based quantification of MM1S cell survival, following 72 h treatment with increasing doses of Dex, in combination with either mock transfection, else transfection with a synthetic mir-150-5p mimetic or mir-150-5p specific antagomir (as indicated in figure). Data represented are relative cell survival levels (mean ± SEM) of three independent experiments. Means with ***, **, * are significantly different (p<0.001, <0.01 or <0.05) from control setup as determined by two-way ANOVA (Bonferroni posttests). (**C**) Realtime quantitative PCR (QPCR) quantification of mir-150-5p transcription levels following 72 h treatment with 1 nM Dex, either or not in combination with transfection of a mir-150-5p specific antagomir. (**D–E**) Illumina BeadChip Gene Expression results of a subset of genes are presented as bargraphs, reflecting the mean fold change from three independent experiments of MM1S cells treated for 72 h with 1 nM Dex vs. control setup, either or not in combination with transfection of a mir-150-5p specific antagomir.

Since mir-150-5p modulates various genes related to cell cycle, cell proliferation and cell death pathways, we next wanted to measure cell death induced by ectopic mir-150-5p transfection. Surprisingly, no substantial induction of cell death could be observed by MTT-assay at 24, 48 and 72 h following transfection of MM1S cells by increasing amounts of mir150-5p (Fig. 10A). The latter suggests that mir-150-5p induced gene expression changes are not sufficient to trigger GC induced cell death and additional GC actions are required to kill the cells [15]. Next, we evaluated whether ectopic mir-150-5p transfection may sensitize for GC induced cell death. In this respect, mock and mir-150-5p transfected MM1S cells were exposed to various doses of Dex for 72 h and cell survival was again evaluated by a MTT-assay. From Fig. 10B, it can be observed that presence of mir-150-5p weakly increases GC induced cell death at nanomolar (nM) concentrations, although no significant effects could be discriminated at micromolar (µM) concentrations of Dex treatment. Moreover, upon GC treatment of MM1S cells transfected with a mir-150-5p specific antagomir, to reverse mir-150-5p effects, cell death could be reversed at low GC doses. This prompted us to evaluate corresponding changes in gene expression following GC treatment of MM1S cells transfected with mir-150-5p antagomir. Although we could confirm weak GC specific upregulation of mir150-5p and mild reduction of mir 150-5p levels upon GC treatment of MM1S cells transfected with mir-150-5p specific antagomir (Fig. 10C), array approaches seemed not sensitive enough to identify weak changes in gene repression or activation in GC treated cells in presence or absence of the antagomir (Fig. 10D–E and data not shown).

## Discussion

Decreased proliferation and induction of cell death by apoptosis are two major cellular events to reduce the growth of and to eliminate cancer cells. GCs are known to induce a cell cycle arrest preceding apoptosis. Treating the cancerous cells with higher doses of Dex can destabilize the glucocorticoid receptor (GR) which ultimately results in GC resistance [38], [81], [82], [83]. The characterization of miRNAs and their target mRNAs involved in regulation of the GC therapy response and/or resistance is an area of intense research and relatively little is known regarding GC specific miRNA regulatory networks. By combining genome-wide transcriptome analysis and Ingenuity Pathway Analysis, we envisioned to unravel the biological effects of GC responsive miRNAs and their putative mRNA targets in MM GC therapy. We identified 30 GC inducible and 14 GC repressed microRNAs which might modulate multiple aspects of GC therapy response in MM, including cell cycle, cell proliferation, DNA replication, gene expression and cell death pathways. Similarly, a study of GC responsive microRNAs in mucosal biopsies of eosinophilic esophagitis patients characterized 32 GC inducible and 4 GC repressed microRNAs, of which 7 GC inducible microRNAs are in common with our list, irrespective of the cellular context [84]. Next, we selected mir-150-5p as the most interesting GC inducible microRNA for further investigation because of its persistent upregulation by GC in MM1S cells and its possible tumor suppressor functions in (malignant) haematopoiesis [26], [54], [85], [86], [87]. According to IPA analysis, predicted mir-150-5p mRNA targets were related to cell cycle and cell proliferation responses preceding apoptosis. In line with IPA analysis, ectopic mir-150-5p expression in presence of low GC concentrations was found to weakly sensitize for GC induced cell death, whereas a mir-150-5p antagomir could reverse this effect. Remarkably, upon ectopic transfection of mir-150-5p or GC treatment in MM1S cells, various mRNAs were identified which respond similarly to increased mir-150-5p levels or GC treatment (i.e. MYB, IL23A, SKP2, BUB1, SREBP1, FKBP5). This suggests that mir-150-5p effects are strongly linked to GR function. Moreover, GR specific mir-150-5p effects are completely lost in MM1R cells and mir-150-5p can not restore GC sensitivity in MM1R cells lacking GR protein expression. Since mir-150-5p overexpression did not substantially change GR expression levels, we believe the GR like effects may be triggered via complex indirect regulation of GR interacting transcription factors (MYB, C/EBP, JUN, FOS) or hormone receptors (NR3C2/MR), GR chaperones (FKBP5, HSPA8, HSP90AB1), as well as various effectors of unfolded protein stress (ATF4, DDIT3, DDIT4, TXNIP) and chemokine specific signalling (CXCR3, CCL3, CCL5, CXCR4). Of special note, mir-150-5p selective decrease in NR3C2 (MR) levels may result in reciprocal stimulation of GR actions [58]. The GR dependent mir-150-5p effects also suggest that GR protein may contribute in mir-150-5p specific post-transcriptional regulation. For example, NR3C1 (GR) dependent post-transcriptional regulation of CCL2 and CCL7 but not of CCL5 mRNA has been demonstrated upon RNA binding of GR to a GU rich motif at the 5′UTR [88]. Alternatively, mir-150-5p may modulate GR/DNA binding, in analogy to the long noncoding RNA Gas5 [24].

Altogether, we identified various novel and previously published GC inducible microRNAs. Furthermore, multiple mir-150-5p responsive mRNAs are associated with GC induced cell proliferation and cell death therapy response in multiple myeloma. Functional cell survival studies in MM1S cells confirmed weak GC therapy sensitizing effects of mir-150-5p overexpression at low GC dose, whereas opposite effects could be observed in presence of a mir-150-5p specific antagomir. However, although QPCR assays revealed reversal of Dex induced mir-150-5p levels following antagomir transfection and attenuation of GC induced cell death, subsequent microarray analysis failed to identify significant mir-150-5p specific changes in GC regulated mRNA levels (Fig. 10D–E and data not shown). We believe that the array study may lack sensitivity and statistical power to identify significant mRNA changes when reversing a 2-fold time-dependent GC specific induction of mir-150p levels by its antagomir, whereas a 30-fold overexpression in mir150-5p levels following transfection may elicit more robust effects in gene expression arrays. In a similar study, failure of antagomirs to reverse mir-92 specific gene expression changes despite reduced levels of mir-92 in presence of its antagomir, was explained by possible redundancy in mir-92 regulated mRNA networks [89]. Along the same line, since we identified various GC inducible microRNAs related to the NR3C1 pathway, we can also not exclude redundant mir-150-5p regulated mRNA networks involved in NR3C1 regulation. For example, early GC inducible microRNAs, i.e. mir-26b, mir125-5p and mir184 could be responsible for subsequent increase of mir150-5p levels to fine tune GR activity (Fig. 3C). Recent reports also show coupling of mir150-5p to mir124- and mir155-networks [90], [91]. Alternatively, redundant mir-150-5p isomirs may escape control of the antagomir [91]. Future research will be required to further untangle specificity or redundancy of mir-150-5p-specific NR3C1 networks by complementary transcriptomic and proteomic approaches. Finally, recent reports demonstrating mir-150-5p secretion into microvesicles may allow development of a diagnostic biomarker to follow GC therapy response or else, combination of low GC doses with synthetic mir-150-5p vesicles may sensitize GC therapy responses in lymphoid cancers [92], [93].

## Supporting Information

Table S1 IPA prediction of microRNA targets based on correlation of transcriptional changes of specific microRNAs (up/down) with changes in mRNA levels (opposite direction down/up) and corresponding pathways in MM1S cells treated for 72 h with Dex.
(XLSX)

Table S2 Crosscomparison of 1203 Dex responsive target genes from MM1S cells exposed for 72 h to 1 µM Dex (gene selection was done based on p-value <0.05 and absolute fold change >1.5) and 3626 predicted hsa-mir-150-5p targets via the miRWalk database (prediction by at least 4 out of 10 available algorithms, i.e. DIANAmT, miRanda, miRDB, miRWalk, RNAhybrid, PICTAR4, PICTAR5, PITA, RNA22 and Targetscan) 196 common mRNA/gene targets were identified in MM1S, which remain unaffected in MM1R cells.
(XLSX)

Table S3 In silico prediction of microRNAs targeting the NR3C1 gene locus versus the NR3C1 3′ UTR.
(XLSX)

Table S4 Detailed list of genes involved in cell cycle-proliferation, DNA repair and cell death pathways which are similarly regulated in Dex treated and mir-150-5p mimetic transfected MM1S cells, but which remain unaffected in MM1R cells (values represent (fold change versus control cells)).
(XLSX)
